# Supplementary material for: Tool Use in Horses
Source: Animals (Basel). 2022 Jul 22;12(15):1876. doi: 10.3390/ani12151876 (PMC9331065; doi:10.3390/ani12151876)
Supplement: Supplementary file 1 [file animals-12-01876-s001.zip › File_S1_Statistical_Data.pdf]

File S1: statistical data

```
Chi-squared test for given probabilities

data:  c(9, 1, 1)
X-squared = 11.636, df = 2, p-value = 0.002973

Warnmeldung:
In chisq.test(c(9, 1, 1)) : Chi-Quadrat-Approximation kann inkorrekt sein
> binom.test(c(9,1))



Exact binomial test

data:  c(9, 1)
number of successes = 9, number of trials = 10, p-value = 0.02148
alternative hypothesis: true probability of success is not equal to 0.5
95 percent confidence interval:
 0.5549839 0.9974714
sample estimates:
probability of success
                   0.9


Spearman's rank correlation rho

data:  category.behaviour.response.to.restriction and category.restriction.
management
S = 83.313, p-value = 0.009877
alternative hypothesis: true rho is not equal to 0
sample estimates:
      rho
0.7086972
```
